# Developing a pharmacist preceptorship programme to support UK advanced level practice: a consensus study

**DOI:** 10.1007/s11096-025-01909-z

**Published:** 2025-04-16

**Authors:** Mairi-Anne McLean, Paul Forsyth, Emma Dunlop, Anne C Boyter

**Affiliations:** 1 Pharmacy Services NHS Greater Glasgow and Clyde, NHS Greater Glasgow and Clyde, Clarkston Court, 56 Busby Road, Clarkston, Glasgow, G76 7AT Scotland, UK; 2 Strathclyde Institute of Pharmacy & Biomedical Sciences, University of Strathclyde, 161 Cathedral St, Glasgow, G4 0RE Scotland, UK; 3 Golden Jubilee University National Hospital, Agamemnon Street, Clydebank, G81 4DY UK

**Keywords:** Advanced practice, Consensus, Pharmacy, Preceptorship

## Abstract

**Background:**

Globally, health professionals are advancing roles to meet growing healthcare demands. Pharmacists are increasingly required to deliver autonomous, holistic, highly complex advanced care. Preceptorship could be used more widely to support delivery of advanced pharmaceutical care.

**Aim:**

The aim of this study was to formulate statements describing features of preceptorship programmes, and to measure consensus in the Scottish pharmacy workforce on the applicability of these statements to an advanced pharmacist preceptorship programme.

**Method:**

Phase 1—formulation of statements relating to key features of healthcare preceptorship programmes through literature review and author expertise. Phase 2—modified nominal group technique (m-NGT) to add expert ideas to phase 1 statements and to reach consensus on statement wording. Phase 3—a two round modified Delphi (m-Delphi) survey to measure consensus in the Scottish pharmacy workforce on whether the features presented should be part of a Scottish advanced pharmacist preceptorship programme. Consensus agreement was set at 75% for m-NGT and m-Delphi.

**Results:**

Fifty-one statements were generated from literature. Seven statements were generated by authors. Three statements were generated by experts during m-NGT stage: 61 statements progressed to m-Delphi.

After two rounds (n = 194 and 144 participants in round one and two respectively) of m-Delphi, consensus was reached on 48 of the 61 statements across categories including programme design, preceptor training requirements and programme assessment.

**Conclusion:**

This study provides a strong basis for research into the impact of preceptorship programmes for pharmacists working towards the advanced career stage.

**Supplementary Information:**

The online version contains supplementary material available at 10.1007/s11096-025-01909-z.

## Impact statements

- The results provide stakeholders with a foundation for the development of preceptorship programmes for pharmacists working towards the advanced career stage.
- Future work with senior pharmacy leaders and educators should place focus on raising awareness and acceptance of the need for advanced pharmacists to have prescribing and leadership skills.

## Introduction

Globally, healthcare faces unprecedented challenges, including demand for high quality healthcare delivery, workforce pressures and increasing spend in healthcare. These challenges have led to skill mix review, role extensions [1], and the International Pharmaceutical Federation (FIP) and World Health Organisation (WHO) urging countries to strengthen capacities of their workforces [2, 3]. Non-medical health professionals are advancing roles to help healthcare systems meet these growing healthcare demands, often raising their scope of practice higher than entry-level requirements and traditional roles [3]. WHO stresses the importance of developing advanced professionals in their 2030 Global Strategy [3] and there is a global shift to include advanced practice in healthcare action plans [4, 5].

In 1997, WHO published the “Seven Star Pharmacist” concept, describing pharmacy practice across a number of domains (care giver, decision maker, communicator, leader, manager, lifelong learner, and teacher) [6]. This vision influenced the development of similar multi-domain advanced pharmacist frameworks, across many different countries and regions [7, 8]. Throughout this paper “advanced pharmacist” is defined as a pharmacist who has been accredited against an advanced practice framework. In principle such pharmacists have the additional knowledge and skills needed to deliver holistic patient-centred care to individuals and/or groups with highly complex needs, including where evidence is limited or ambiguous, autonomously managing risk in the presence of significant uncertainty [8]. They will be able to use a range of assessment methods, including clinical examination and other skills, adapting both these and communication style based on the individual [8].

Advanced pharmacist frameworks describe the delivery of care with a higher degree of autonomy for people with more complex needs, often requiring pharmacists to deviate from protocol or guideline driven care [7, 8]. As an added layer of responsibility and accountability, pharmacists across the globe are also increasingly becoming credentialed as independent prescribers [9], a role extension where pharmacists have encountered barriers [9], including risk aversion, low self-confidence, and difficulty achieving support while enablers include strong support networks and coordinated organisational support [10–12]. If pharmacists are to confidently and competently deliver this extended autonomous care, there is recognition that advanced pharmacists need support to flourish [13], however the precise nature of effective support is yet to be established. Frameworks, such as the FIP’s Global Advanced Development Framework and the Royal Pharmaceutical Society (RPS) Core Advanced Curriculum [7, 8], support pharmacists by defining the required outcomes. These frameworks focus on evidencing the ability to autonomously care for complex patients while managing risk and uncertainty, supervised by more experienced competent multidisciplinary peers and colleagues.

Further support that may deliver these outcomes in practice, specifically to improve pharmacist confidence and competence in the advanced career stage is preceptorship, a form of clinical supervision [14]. Healthcare preceptorship programmes were developed in North America in the 1970s. Pharmacist preceptorship programmes are common in United States of America (USA) and Canada, particularly during student and early career stages [15], although their application elsewhere is limited. Preceptorship is also embraced in other healthcare professions such as nursing, as a means of improving functionality and skill especially at career transition points [16]. Preceptorship provides structured support with the aim of integrating professionals into team culture while translating knowledge into practice [17]. Widespread acknowledgment of the benefits of preceptorship, including improved confidence, sense of belonging, and professional identity [18] has led to an increase in the number of countries and professions offering this form of clinical supervision. Preceptorship for pharmacists working towards this more complex and autonomous advanced level practice could build self-confidence and competence, thus improving delivery of care to patients.

### Aim

The aim of this study was to formulate statements that describe features of preceptorship programmes, and to measure consensus, in the Scottish pharmacy workforce, on the applicability of these statements to a future advanced pharmacist preceptorship programme.

### Ethics approval

Ethics assessment was completed by the Strathclyde Institute of Pharmacy and Biomedical Sciences, University of Strathclyde on 29th March 2022. Full ethics approval was not required as the project was service evaluation.

## Method

### Study setting

The study was set in National Health Service (NHS) Scotland. NHS Scotland consists of 14 regional health authorities, and eight additional organisations which provide specialist (e.g. public health, education etc.) and national services. The NHS is funded through taxation and is, for most services, free at the point of delivery [19]. Scotland has a population of around 5.5 million [20].

### Positionality

The first author is a white, middle class, female, non-patient facing, pharmacist prescriber (non-active) with 27 years of experience in primary care and community pharmacy and three years of experience as a research fellow. She has experienced and observed the challenges facing pharmacists attempting to transition into advanced roles. The senior author is a white, middle class, female in an academic role. She has 39 years of experience initially as a hospital pharmacist and for the last 28 years in academia in a School of Pharmacy. As a result, she is familiar with pharmacy practice and the pedagogy underpinning preceptorship. The second author is a white, middle class, male, patient facing, pharmacist prescriber (active) with 22 years of experience in primary care, community pharmacy and hospital outpatient clinics. His academic and educational interests include advanced pharmacy practice.

The third author is a white, middle class, female researcher with 15 years of experience in health service research in a School of Pharmacy. She has a familiarity with some of the issues facing pharmacists through previous research projects.

### Study design summary

The study comprised three methodological phases: 1) statement formulation from literature and authors expertise, 2) finalising a comprehensive list of appropriately worded statements through a modified Nominal Group technique (m-NGT), and 3) workforce-wide consensus measurement through a modified Delphi (m-Delphi).

### Phase 1: formulation of statements

#### **Aim:**

 Phase 1 aimed to formulate a comprehensive list of statements that each related to a key feature of a healthcare preceptorship programme.

#### **Rationale:**

 The comprehensive list of appropriately worded statements would allow voting in a future m-Delphi.

#### **Design:**

 Statements were formulated through a comprehensive literature search relating to preceptorship programme design and through expert review by the authors.

#### **Data sources and dates:**

 EMBASE and MEDLINE databases were searched, from inception to February 2022, to identify literature describing core features of preceptorship programmes. Key search terms included “preceptorship”, “programme”, “pharmacist”, “confidence” and “competence” (see supplementary files for further detail of search terms, search structure and data extraction). Grey literature sources, such as Google (first 50 hits), books [15, 16], and references from academic opinion pieces were searched, to improve completeness of the search.

The authors also used their knowledge of the subject to add pharmacy specific statements.

#### **Inclusion or exclusion criteria:**

Literature included described features of healthcare preceptorship programmes. Literature of all types and from all countries was included. Non-English language studies were excluded.

#### **Data extraction:**

 Data relating to the core features of healthcare professional preceptorship programmes was extracted by the lead author (MMcL) and collated in Microsoft Word^©^.

#### **Statement formulation:**

 Features of healthcare preceptorship programmes found in the literature were inductively sorted into categories and cross referenced. This complete list was screened by two authors with experience of advanced pharmacist practice. One author (MMcL) sorted the features into categories and drafted a statement relating to each feature and the second (PF) verified the approach to categorisation and initial statement wording. The two authors also used their experience of preceptorship and advanced practice to add pharmacy specific statements from pharmacist career frameworks [8, 21, 22], strategic documents [23–25] and experience. Statements were worded to allow rating against a 5-point Likert scale in Phase 3 (m-Delphi). The formulated statements spanned eight categories of programme design, preceptor capabilities and experience, preceptor qualifications, preceptor training requirements, preceptor qualities and behaviours, preceptee characteristics, programme assessment and outcome measures, and programme follow up.

### Phase 2: expert review of statement wording and additional statement generation (m-NGT)

#### **Aim:**

 The aim of phase 2 was two-fold: 1) to allow experts to add to the literature derived statements from phase 1 and 2) reach expert consensus on the wording of statements.

#### **Rationale:**

 Expert review of statements was required to ensure m-Delphi statements were comprehensive and appropriately worded for the intended audience.

#### **Design:**

 A modified Nominal Group Technique (m-NGT), comprising a phase 1 statement wording review exercise (the modification) plus standard four-stage NGT (silent generation, round robin, clarification and voting) [26, 27].

An online questionnaire (questionnaire one) was sent to each participating expert in advance of an online meeting. Questionnaire one presented each statement from phase 1 and experts were asked to vote “yes/no” on whether wording was appropriate for a future m-Delphi. If voted as not appropriately worded, alternative wording could be suggested. Questionnaire one also captured “silent idea generation” ideas by allowing the experts to enter free text suggestions for additional statements. Questionnaire one was completed in advance, to reduce the length of the online meeting. Participants were asked to return the completed questionnaire one within two weeks. The lead author collated results from expert opinions on statement wording and “silent idea generation” ideas (i.e. questionnaire one). A final voting questionnaire (questionnaire two), for use during the online m-NGT, was then prepared.

The remaining standard NGT stages (round robin, clarification and voting) were conducted during an online meeting. The online meeting was recorded with auto transcription.

#### **Participant inclusion or exclusion criteria:**

 The aim was to recruit four participants (sample size based on literature and convenience) [27], who among them had experience of the following criteria a) delivering advanced practice / completing an advanced framework, b) delivering advanced clinical supervision, c) creating advanced strategic policy and/or d) being a current advanced pharmacist learner (See supplementary files for further information). Apart from the learner criteria, these criteria were not mutually exclusive.

#### **Study material development:**

 A participant information sheet, participant consent form, and two questionnaires were developed. Questionnaire one was piloted by two University of Strathclyde researchers with minor amendments. Both m-NGT questionnaires were hosted on Qualtrics^©^ [Version 2022]**.**

#### **Participant recruitment:**

A mixed approach was used to recruit four experts. Purposeful sampling was used to identify expert participants who met the above criteria. Potential participants were then approached through professional email networks in March 2022. All participants accepted the first invitation and were provided with participant information.

#### **Data collection:**

 In April 2022, participants were sent questionnaire one and then invited to an on-line (Microsoft Teams®) m-NGT on 19 May 2022. Completion of questionnaire one was stage 1 of the m-NGT. During the online meeting, chaired by the lead researcher, results and ideas from the m-NGT questionnaire were displayed in an MS PowerPoint® presentation. During stage 2, “clarification”, individuals presented their ideas for new statements or revisions of any Stage 1 literature derived statements. Individual presentation (round robin) was followed by facilitated group discussion. A facilitator edited the prepared questionnaire two as revisions and additions were presented. Stage 3 “voting” presented questionnaire two (original wording of statements, reworded statements and statements added by the experts). Statements in questionnaire two were presented in two sections (A and B). Section A presented statements added or modified during the meeting along with original wordings. Where more than one version of wording was presented, experts selected the version they felt was most appropriate. Section B re-presented statements that had achieved consensus in questionnaire one. None of these statements had re-wording suggested. These statements were presented again to ensure that opinions had not changed after stage 2 discussions. In Stage 4, the lead researcher fed back the results of the final voting to the experts.

#### **Data analysis:**

 Consensus was set *a-priori* at 75% agreement as between 70 and 80% is suggested in the literature [28] and 75% would reflect agreement by three of four experts. Data were extracted into Microsoft Excel^©^. Questionnaire one data contained yes/no responses and free text. Data were used to prepare the Questionnaire two. Questionnaire two data were analysed for consensus.

### Phase 3: consensus measurement—modified Delphi

#### **Aim:**

 Phase 3 aimed to measure consensus in the Scottish pharmacy workforce on whether the features presented should be part of a Scottish advanced pharmacist preceptorship programme.

#### **Rationale:**

 Professional consensus is required to design a future pilot study to report on the impact of preceptorship on pharmacists working towards the advanced career stage.

#### **Design:**

 The study design was modified from standard Delphi in two ways. Firstly, participants were not recruited. Pharmacists were sent the surveys and were free to choose whether to complete or not. Secondly, only statements that had failed to reach consensus in round 1 were included in round 2. The two-round m-Delphi survey comprising statements from phase 2 (m-NGT) was disseminated to pharmacists in Scotland. Views on statements were captured on a 5-point Likert scale (Strongly agree, agree, neutral, disagree, and strongly disagree). M-Delphi round 1 included all phase 2 statements. After m-Delphi round 1, results were analysed to determine which statements had met consensus. In m-Delphi round 2, participants were presented with statements (unchanged from round 1) that had not achieved consensus agreement alongside the results from round 1.

#### **Participants inclusion or exclusion criteria:**

 Eligible participants were all pharmacists (n = 2119 [29]) employed by NHS Scotland organisations with patient-facing and/or educational roles (Scottish regional NHS Health Boards, NHS Education for Scotland, NHS 24, NHS Golden Jubilee University National Hospital and/or State Hospitals Board for Scotland). Sample size for m-Delphi was not predetermined in order to maximise sample population. All pharmacists would have experienced clinical supervision during their early-career training and therefore had the required knowledge to respond [30].

#### **Study material development:**

 The surveys, hosted on Qualtrics^©^ [Version 2022], included participant information and survey details. The surveys were piloted by three individuals who had experience of pharmacist advanced practice but who fell outside the inclusion criteria. Piloting led to minor amendments.

#### **Participant recruitment:**

 M-Delphi round 1 was cascaded through national professional networks (Directors of Pharmacy, National Acute Pharmacy (NAPs) group, Scottish Practice Pharmacist and Prescribing Advisers Association (SP3AA) and NHS Education for Scotland—Pharmacy) and social media (X) in June 2022. M-Delphi round 2 was cascaded by the same method in August 2022. Each m-Delphi round remained open for four weeks and a reminder was cascaded after two weeks.

#### **Data collection:**

 The survey collected participant consent, demographic information and participant’s views on how appropriate each statement was for an advanced pharmacist professional practice preceptorship programme.

#### **Data analysis:**

 In line with the m-NGT consensus agreement was defined *a-priori* as > 75% of respondents answering either ‘agree’ or ‘strongly agree’. The data were exported from Qualtrics^©^ into Microsoft Excel^©^. Partial responses were removed, and numerical values were assigned to each Likert response. Formulae in Microsoft Excel^©^ were used to calculate percentages.

## Results

### Phase 1: formulation of statements

The literature search identified 20 suitable sources of information about preceptorship programmes. From these sources, 51 statements were formulated and a further 7 statements were added by the research team, making 58 in total (For statement origins including references see supplementary file).

### Phase 2: expert review of statements

From questionnaire one, consensus was met on the wording of 40 of the 58 phase 1 statements. After idea generation, discussion and clarification, experts generated three additional statements and proposed wording. New wording was suggested for the 18 statements that had not met consensus. Sixty-one statements progressed to final voting. Consensus was met on the wording for all 61.

### Phase 3: consensus measurement

#### Modified Delphi survey

The m-Delphi had a response rate of 9% (194/2119) in round 1 and 7% (144/2119) in round 2. Participant demographics were broadly similar across Round 1 and 2, 75% vs 79% female, 18 years vs 20 years median years registered, and 40% vs 39.5% hospital-based respondents respectively (see Table 1).

**Table 1:** Delphi study round 1 and 2 participant demographics

	Delphi round one (n = 194)	Delphi round two (n = 144)
	N (%)	N (%)
*Gender*		
Female	146 (75%)	114 (79%)
Male	45 (23%)	29 (20%)
Non-binary	1 (0.5%)	1 (0.7%)
Prefer not to say	2 (1%)	0
*Ethnicity*		
White British	169 (87%)	126 (87.5%)
White other	9 (5%)	7 (4.8%)
Asian/Asian British	5 (2.5%)	4 (2.8%)
Mixed	2 (1%)	3 (2.1%)
Arab	0	1 (0.7%)
Black or Black British	0	1 (0.7%)
Other	6 (3%)	0
Prefer not to say	3 (1.5%)	2 (1.4%)
*Years Registered*		
Median (IQR1, IQR3)	18 (10,25)	20 (12, 24.5)
AfC* Banding		
6	3 (1.5%)	4 (2.7%)
7	54 (28%)	36 (25%)
8A	89 (46%)	73 (51%)
8B	32 (16%)	19 (13.2%)
8C	9 (5%)	7 (4.8%)
8D	4 (2%)	4 (2.7%)
9	3 (1.5%)	1 (0.7%)
*Sector of work*		
Acute Care (hospital)	76 (40%)	57 (39.5%)
Primary Care	86 (44%)	60 (41.7%)
Other	32 (16%)	27 (18.7%)
Primary work setting**		
Rural	36 (19%)	24 (17%)
Urban	158 (81%)	120 (83%)
*Line manager of AfC Band 7 pharmacist or above?*		
Yes	93 (48%)	64 (44%)
No	101 (52%)	80 (56%)

*Agenda for change is the main pay system for staff in the NHS, except doctors, dentists and senior managers. Abbreviated to AfC and also known as NHS Terms and Conditions of Service

**Participants self-identified as working in predominantly rural or urban setting

**Fig. 1:**
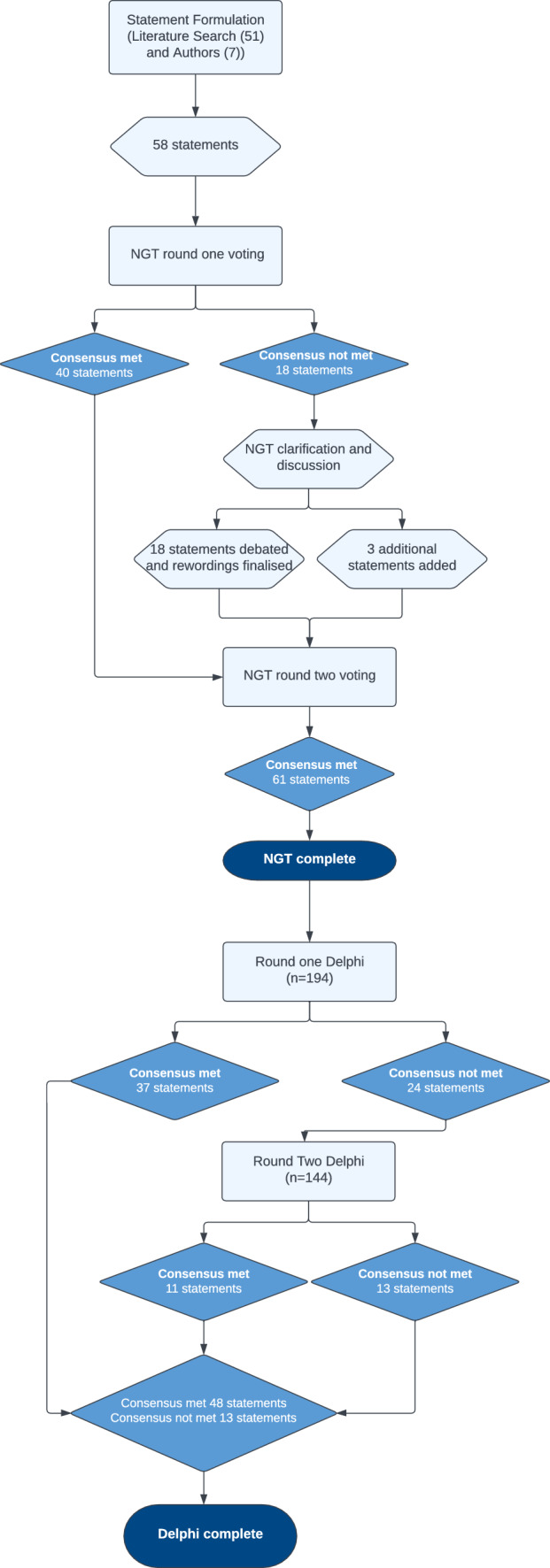
Visual of the flow of consensus method

In m-Delphi round 1, 37 of the 61 statements across eight categories reached consensus agreement. In round 2, 11 of the remaining 24 statements reached consensus agreement. After 2 rounds, 48 of 61 statements reached consensus (19/26 statements on programme design, 5/5 on preceptor capabilities and experience, 0/2 on preceptor qualifications, 2/3 on preceptor training requirements, 6/6 on preceptor qualities and behaviours, 8/10 on preceptee characteristics, 6/7 on programme assessment and outcome measures, and 2/2 on programme follow up). Table 2 shows full results and the wording of every statement, including whether consensus was achieved or not. Figure 1 shows the entire flow of consensus development from phase 1 to the end of phase 3.

**Table 2:** Delphi round 1 and 2 results

Statement	Delphi 1 (n = 194)				Delphi 2 (n = 144)			
	Agree*	Neutral	Disagree**	Consensus Reached?	Agree*	Neutral	Disagree**	Consensus Reached?
*Section one—programme design*								
The advanced career stage preceptorship programme is applicable to pharmacists who have newly started training towards the advanced career stage	172 (88.7%)	16 (8.2%)	6 (3.1%)	✔	N/A			
The advanced career stage preceptorship programme is applicable to pharmacists who are returning to advanced career stage after a career break	162 (83.5%)	24 (12.4%)	8 (4.1%)	✔	N/A			
The advanced career stage preceptorship programme is required for pharmacists who are switching from one advanced specialty to another	133 (68.6%)	39 (20.1%)	22 (11.3%)	X	112 (77.8%)	24 (16.7%)	8 (5.6%)	✔
The start of the preceptorship programme should align with entry into training towards the advanced career stage	161 (83.0%)	19 (9.8%)	14 (7.2%)	✔	N/A			
The advanced career stage preceptorship programme should only be available to pharmacists who are qualified prescribers	65 (33.5%)	24 (12.4%)	105 (54.1%)	X	33 (22.9%)	7 (4.9%)	104 (72.2%)	X
The core learning outcomes for the preceptorship programme should be drawn from the outcomes of the RPS Advanced Pharmacist Curriculum	161 (83.0%)	26 (13.4%)	7 (3.6%)	✔	N/A			
Any additional agreed learning outcomes should be sourced from alternative specialist frameworks	133 (68.6%)	44 (22.7%)	17 (8.7%)	X	88 (61.1%)	47 (32.6%)	9 (6.3%)	X
An individual learning plan to meet and evidence the learning outcomes should be agreed between the preceptor and the preceptee	188 (96.9%)	6 (3.1%)	0 (0.0%)	✔	N/A			
*Completion of a preceptorship programme should take approximately how long? (participants selected one option that they thought most appropriate)*								
4 months ***	26 (13.4%)	73 (37.6%)	95 (49.0%)	X				
6 month ***	53 (27.3%)	78 (40.2%)	63 (32.5%)	X				
12 months ***	60 (30.9%)	88 (45.4%)	46 (23.7%)	X				
Flexible depending on the individual	181 (93.3%)	5 (2.6%)	8 (4.1%)	✔				
The preceptor and preceptee should share a primary workplace (advanced clinical setting)	128 (66.0%)	44 (22.7%)	22 (11.3%)	X	99 (68.8%)	32 (22.2%)	13 (9%)	X
The preceptor and preceptee should work alongside each other regularly allowing observation of work tasks	145 (74.7%)	32 (16.5%)	17 (8.8%)	X	111 (77.1%)	25 (17.4%)	8 (5.6%)	✔
A preceptorship programme should include regular review meetings	189 (97.4%)	5 (2.6%)	0 (0.0%)	✔	N/A			
A preceptorship programme should include a preceptee training pack	143 (73.7%)	42 (21.6%)	9 (4.6%)	X	120 (83.3%)	17 (11.8%)	7 (4.9%)	✔
A preceptorship programme should include preceptee workshops	126 (64.9%)	57 (29.4%)	11 (5.7%)	X	95 (66%)	40 (27.8%)	9 (6.2%)	X
A preceptorship programme should include preceptee protected learning time	182 (93.8%)	10 (5.2%)	2 (1.0%)	✔	N/A			
A preceptorship programme should include preceptor protected learning time	178 (91.8%)	14 (7.2%)	2 (1.0%)	✔	N/A			
The employing organisation should ensure preceptor and preceptees protected time	182 (93.8%)	9 (4.6%)	3 (1.5%)	✔	N/A			
A preceptorship programme should include a preceptor policy within your organisation	166 (85.6%)	25 (12.9%)	3 (1.5%)	✔	N/A			
A preceptorship programme should be supported by organisational preceptor support infrastructure	170 (87.6%)	22 (11.3%)	2 (1.0%)	✔	N/A			
A preceptorship programme should include organisational level preceptor lead	134 (69.1%)	52 (26.8%)	8 (4.1%)	X	113 (78.5%)	27 (18.8%)	4 (2.7%)	✔
A preceptorship programme should include a learning contract	132 (68%)	47 (24.2%)	15 (7.7%)	X	99 (68.8%)	30 (20.8%)	15 (10.4%)	X
A preceptorship programme should include learning diaries	82 (42.3%)	85 (43.8%)	27 (13.9%)	X	36 (25%)	83 (57.6%)	25 (17.4%)	X
A preceptorship programme should include a requirement for the preceptee to undertake significant event analysis or equivalent	128 (66.0%)	42 (21.6%)	24 (12.4%)	X	89 (61.8%)	31 (21.5%)	24 (16.7%)	X
A preceptorship programme should include activities designed to induct the preceptee into the advanced practice environment and team	182 (93.8%)	9 (4.6%)	3 (1.5%)	✔	N/A			
A preceptorship programme should include activities designed to integrate pharmacists training towards the advanced career stage into the culture of the advanced practice environment	178 (91.8%)	15 (7.7%)	1 (0.5%)	✔	N/A			
The preceptorship programme must be quality managed by a nominated body	139 (71.6%)	46 (23.7%)	9 (4.6%)	X	116 (80.6%)	23 (16%)	5 (3.4%)	✔
*Section two—preceptor capabilities/experience*								
Preceptors should be able to demonstrate relevant experience at advance practice level	188 (96.9%)	4 (2.1%)	2 (1%)	✔	N/A			
Preceptors should be able to demonstrate competence at the advanced practice level or above in a relevant clinical area	182 (93.8%)	6 (3.1%)	6 (3.1%)	✔	N/A			
Preceptors should be able to demonstrate confidence in the delivery of advanced practice	187 (96.4%)	3 (1.5%)	4 (2.1%)	✔	N/A			
Preceptors should be able to demonstrate relevant experience of teaching clinical practice	144 (74.2%)	41 (21.1%)	9 (4.6%)	X	120 (83.3%)	17 (11.8%)	7 (4.9%)	✔
Preceptors should be able to demonstrate current delivery of clinical practice at the advanced practice level or above	174 (89.7%)	12 (6.2%)	8 (4.1%)	✔	N/A			
*Section three—preceptor qualifications*								
The preceptor should be a registered pharmacist	101 (52.1%)	62 (31.9%)	31 (16%)	X	60 (41.7%)	61 (42.3%)	23 (16%)	X
The preceptor should be any appropriate healthcare colleague	115 (59.3%)	34 (17.5%)	45 (23.2%)	X	103 (71.5%)	13 (9%)	28 (19.5%)	X
*Section four—preceptor training*								
In order to undertake the role of preceptor, the individual will need to undertake specific training relating to the programme	127 (65.5%)	47 (24.2%)	20 (10.3%)	X	116 (80.6%)	15 (10.4%)	13 (9%)	✔
Preceptors should not need to undertake specific training other than familiarisation with the advanced pharmacist curriculum and programme materials	80 (41.2%)	23 (11.9%)	91 (46.9%)	X	43 (29.9%)	14 (9.7%)	87 (60.4%)	X
Preceptors will need to undertake a regular peer review process of their preceptorship skills	139 (71.6%)	37 (19.1%)	18 (9.3%)	X	116 (80.6%)	19 (13.2%)	9 (6.2%)	✔
*Section five—preceptor qualities and behaviours*								
Preceptors should be able to demonstrate willingness to undertake the preceptor role	187 (96.4%)	5 (2.6%)	2 (1%)	✔	N/A			
Preceptors should be able to demonstrate characteristics of a role model	185 (95.4%)	7 (3.6%)	2 (1%)	✔	N/A			
Preceptors should be able to demonstrate evidence of person centred practice	187 (96.4%)	5 (2.6%)	2 (1%)	✔	N/A			
Preceptors should be able to demonstrate excellent interpersonal skills	185 (95.4%)	8 (4.1%)	1 (0.5%)	✔	N/A			
Preceptors should be able to demonstrate an understanding of the skill level of pharmacists training towards the advanced career stage	191 (98.5%)	2 (1%)	1 (0.5%)	✔	N/A			
Preceptors should be able to demonstrate an understanding of the preceptor/preceptee relationship and the preceptorship programme purpose	188 (96.9%)	4 (2.1%)	2 (1%)	✔	N/A			
*Section six—preceptee characteristics*								
Preceptees (learners) should be able to demonstrate self-motivation	183 (94.3%)	9 (4.6%)	2 (1%)	✔	N/A			
Preceptees should be able to demonstrate active participation	184 (94.8%)	7 (3.6%)	3 (1.5%)	✔	N/A			
Preceptees should be able to demonstrate evidence of person centred practice	170 (87.6%)	16 (8.2%)	8 (4.1%)	✔	N/A			
Preceptees should be able to demonstrate the ability to relate current experience to previous experience	166 (85.6%)	27 (13.9%)	1 (0.5%)	✔	N/A			
Preceptees should be able to demonstrate leadership skills	120 (61.9%)	54 (27.8%)	20 (10.3%)	X	78 (54.2%)	42 (29.2%)	24 (16.6%)	X
Preceptees should be able to demonstrate initiative	176 (90.7%)	14 (7.2%)	4 (2.1%)	✔	N/A			
Preceptees should be able to demonstrate tolerance of ambiguity	123 (63.4%)	56 (28.9%)	15 (7.7%)	X	95 (66%)	44 (30.6%)	5 (3.4%)	X
Preceptees should be able to demonstrate maturity	158 (81.4%)	29 (14.9%)	7 (3.6%)	✔	N/A			
Preceptees should be able to demonstrate professionalism	189 (97.4%)	4 (2.1%)	1 (0.5%)	✔	N/A			
Preceptees should be able to demonstrate good judgement	182 (93.8%)	10 (5.2%)	2 (1%)	✔	N/A			
*Section seven—assessment/outcome measures*								
Preceptees should be assessed by review of a portfolio of evidence	145 (74.7%)	36 (18.6%)	13 (6.7%)	X	123 (85.4%)	14 (9.7%)	7 (4.9%)	✔
Preceptees should be assessed against competency outcomes	174 (89.7%)	13 (6.7%)	7 (3.6%)	✔	N/A			
Preceptees should be assessed for their ability to deliver person centred practice	181 (93.3%)	11 (5.7%)	2 (1%)	✔	N/A			
Preceptees should be assessed against completion of supervised learning events	160 (82.5%)	28 (14.4%)	6 (3.1%)	✔	N/A			
Preceptees should be assessed by submission of reflective account(s)	146 (75.3%)	34 (17.5%)	14 (7.2%)	✔	N/A			
Preceptees should be assessed by means of a formalised clinical skills assessment appropriate to advanced role	146 (75.3%)	36 (18.6%)	12 (6.2%)	✔	N/A			
Preceptees should be assessed by means of a knowledge test	70 (36.1%)	54 (27.8%)	70 (36.1%)	X	21 (14.6%)	50 (34.7%)	73 (50.7%)	X
*Section eight—programme follow up*								
At the end of the Preceptorship programme, support will be continued remotely for example by email or professional conversations	145 (74.7%)	35 (18%)	14 (7.2%)	X	120 (83.3%)	18 (12.5%)	6 (4.2%)	✔
At the end of the Preceptorship programme, support will be continued through standard clinical supervision channels	139 (71.6%)	50 (25.8%)	5 (2.6%)	X	115 (79.9%)	25 (17.4%)	4 (2.7%)	✔

N/A, Not Applicable. Statements that reached consensus in round one were excluded from round two and so there are no round two results

*Amalgamated “Agree” and “Strongly Agree” responses in line with consensus level pre-agreed at study design

**Amalgamated “Disagree” and “Strongly Disagree” responses in line with consensus level pre-agreed at study design

***Consensus not met in round one but removed as interdependencies with statement which reached consensus in round one

## Discussion

### Summary of key findings

This study found high levels of consensus on features derived from the literature and expert panel relating to a Scottish advanced pharmacist preceptorship programme. Consensus was reached on 48 out of 61 statements.

### Strengths and weaknesses

#### Strengths

This is the first study to explore pharmacist views on features of an advanced pharmacist preceptorship programme using robust consensus methods. The two-stage (m-NGT plus m-Delphi) consensus study added to the rigour of results. The reporting of this study adheres to the reporting standards for Delphi [31]. Participant characteristics in the m-Delphi were broadly similar across both rounds and generally representative of the pharmacist workforce (e.g. more female than male participants and the proportion of primary care and hospital-based participants as expected) [32, 33].

#### Weaknesses

Limitations in methodology include a lack of rigour to search of grey literature (e.g. potential for relevant sources to have been missed), no independent double screening and time elapsed between review and publication. Statements formulated in phase 1 did not undergo independent double review. Participants in m-NGT and m-Delphi were from a single country and from within the NHS only, which may limit generalisability. NGT experts were identified through a mixed approach which included identification through convenience which could potentially introduce bias. There was potential for statements to be interpreted differently depending on m-NGT and m-Delphi participant’s knowledge and experience which may have influenced responses. Sample size was not predetermined for m-Delphi. The two rounds of m-Delphi responders were potentially different responders which could have influenced results despite similar demographics across the two rounds. The potential for bias in the m-Delphi was minimised through careful wording of statements, piloting of questionnaires, large sample of m-Delphi participants and participant demographics generally representative of the pharmacist workforce. Despite the steps taken to minimise bias in the m-Delphi, potential for bias remains and is noted as a weakness. The subject matter of advanced practice may not be well understood, as it is not professionally regulated [34]. Delphi response rates were low (9% and 7%); however, this response rate was anticipated, due to number of invited participants, survey length and complexity, survey distribution constraints, level of emails received by this professional group and the time required to participate [35]. For these reasons, the response rate across such a large nation-wide sample was considered to be acceptable. Delphi results have been analysed quantitatively only.

### Interpretation

Consensus was met on all statements about preceptor capabilities/experience, preceptor qualities and behaviours, and programme follow up. As described in results, there was broad agreement on sections relating to preceptor training and programme assessment and outcome measures. As statements had their roots in literature, this shows participant views align with current delivery of preceptorship. As the statements in these categories may been viewed as easier to implement, this may have influenced acceptability of these statements to participants.

No consensus was reached on preceptor professional background: whether the preceptor needs to be a pharmacist or another healthcare professional. When preceptors are of the same profession, there can be benefits of more in-depth knowledge and familiarity with the role [36]. Deeper discussion and guidance can take place, and preceptors understand the skill level expected, so pharmacists may gain maximum benefit from preceptorship by another pharmacist. As seen in other countries, there is a need to develop a structured approach to the delivery of preceptor training strategies [37] and there may be a lack of availability of pharmacists working at an appropriate level to be preceptors [14]. These challenges, such as identifying adequate numbers of suitably qualified preceptors and releasing them from other duties may have impacted how participants responded and ultimately on lack of consensus on pharmacists being identified as preceptors. Scottish pharmacists may also be more familiar with role modelling delivered by doctors, as historically doctors have supervised pharmacists through independent prescribing qualifications[21, 38]. However, preceptors from different professions describe a lack of awareness of preceptees role and scope of practice, acknowledging this impacts on ability to precept fully [36].

Lack of consensus on the requirement for pharmacist preceptees to be independent prescribers to begin a clinical advanced pharmacist preceptorship programme is an important finding. This seems to be at odds with the preceptee curriculum, which states that pharmacists need to demonstrate that they “*autonomously make appropriate clinical decisions and prescribing interventions*”[8]. All newly qualified pharmacists in the UK will become prescribers at qualification from 2026 [23] and the professional vision in Scotland is that all patient-facing pharmacists will be independent prescribers by 2030 [25]. Barriers to pharmacists prescribing, where implemented across the world, are well described, including poor awareness of pharmacist prescribing, lack of support from employers, the multidisciplinary team and the public, and difficulties with decision making [9]. Globally, non-medical prescribers, including pharmacists, describe challenges to prescribing including administrative issues, lack of confidence, lack of clinic space and inadequate support mechanisms [39] that may contribute to professional hesitance to embrace prescribing as an essential element of an advanced pharmacist preceptorship programme. There is evidence which supports that practicing pharmacists do not universally view themselves as clinicians, and do not identify with the delivery of patient facing clinical practice [40], which may support the results of this study.

The requirement for preceptees to demonstrate leadership skills at the start of the programme also did not reach consensus. Leadership and management are key elements in both the foundation curriculum that precedes advanced practice [41] and the national regulatory standards for pharmacists [42]. Ambiguity is known to exist regarding the concept and competencies required to deliver pharmacist leadership [43] and the underdevelopment of leaders in pharmacy is a barrier to both curriculum completion [44] and advancement of the profession [45]. There is a need to address the barriers to leadership development of pharmacists, if the profession is to achieve its ambitions of population-level clinical pharmacy service expansion [45, 46].

### Further research

Understanding why there is a lack of consensus among pharmacists that those working towards the advanced career stage require to be independent prescribers is a priority if strategic goals to deliver autonomous care to patients are to be met [47]. Further exploration of views on leadership as a core skill [47] for pharmacists at all career stages is needed to provide insight into the lack of consensus on leaderships skills for those working towards the advanced career stage. Exploring lack of consensus in prescriber status and leadership could be undertaken through qualitative methodology, underpinned by an appropriate theoretical model such as normalisation process theory. Further research into the impact of preceptorship programmes on pharmacists working towards advanced level will inform the development of education and training in this area and will allow expansion of pharmacist preceptorship across the UK. A pilot evaluation of the feasibility of a preceptorship programme developed from the results of paper is now underway in Scotland, before the eventual testing of its effect size in a larger and more definitive study.

## Conclusion

Pharmacists need confidence to transition from traditional practice into advanced roles. Preceptorship can increase confidence and competence but has not been studied widely in pharmacists at the advanced career stage.

Study participant views align with current delivery of preceptorship providing a strong basis for research into the impact of preceptorship programmes for pharmacists working towards the advanced career stage. A strategy to raise awareness and acceptance of the need for advanced pharmacists to have prescribing and leadership skills may support the integration of multi-domain advanced pharmacist practice.

## Supplementary Information

Below is the link to the electronic supplementary material.

Supplementary file1 (DOCX 78 KB)
