# Supplementary material for: Developing a pharmacist preceptorship programme to support UK advanced level practice: a consensus study
Source: Int J Clin Pharm. 2025 Apr 16;47(5):1248–60. doi: 10.1007/s11096-025-01909-z (PMC12432034; doi:10.1007/s11096-025-01909-z)
Supplement: Supplementary file 1 — Supplementary file1 (DOCX 78 KB) [file 11096_2025_1909_MOESM1_ESM.docx]

**Supplementary Files**

1. **Literature Search Terms**

| **Search terms** | |
| --- | --- |
| Preceptorship AND programme AND structure:  Pharmacists AND (clinical competence OR professional competence) AND (preceptorship OR preceptor OR mentor* OR clinical supervision):  Preceptor* AND professional competence:  Preceptor* AND professional competence AND confidence:  Preceptor* AND professional autonomy:  Clinical competence OR professional competence OR confidence OR decision making AND Preceptor* | |
| **Data extracted** | |
| Authors and year of publication, title, methods, results, conclusions, elements of preceptorship programme | |
| **Eligibility criteria for study selection** | |
| Inclusion & exclusion criteria | Rationale |
| Inclusion  Papers that describe detail on implemented preceptorship programmes where the following programme elements were described in study (programme design, preceptor capabilities and experience, preceptor qualifications, preceptor training requirements, preceptor qualities and behaviours, preceptee characteristics, programme assessment and outcome measures, and programme follow up).  Papers were excluded where programme elements could not be identified | Literature will be used to synthesise statements that describe the core features of preceptorship programmes |
| Context:  Studies from all countries included Sector: All sectors of practice, all levels or care (primary/secondary/tertiary) included | All relevant areas and settings were included to ensure inclusion of a broad base of global evidence. |
| Language:  Only English language studies included (all returned articles were in English). | Only English language studies included for ease of understanding |
| Data range:  Up to Feb 2022 | To broaden the search and retrieve all relevant papers |
| Types of study:  Full text published papers including primary studies, systematic reviews, scoping reviews and narrative reviews. Government publications and professional grey literature. | To broaden the search and retrieve all relevant information. |

1. **Nominal Group Technique Experts**

Recruitment took place for the NGT to ensure a group of advanced practice experts who among them had;

- Criterion 1 - Contemporary experience of delivering advanced or consultant level practice, including undertaking completion of an advanced competence framework
- Criterion 2 - Experience of delivering clinical supervision at advanced level
- Criterion 3 – Expertise of creating strategic policy or national programmes for advanced practice
- Criterion 4 – the perspective of relevant learner (a pharmacist ready to undertake RPS Advance Pharmacy Practice Curriculum)

|  | Number of Participants meeting this criterion |
| --- | --- |
| Criterion 1 | 2 |
| Criterion 2 | 3 |
| Criterion 3 | 3 |
| Criterion 4 | 1 |

1. **Delphi Statement Origins**

| **Statement** | **Reference** |
| --- | --- |
|  |  |
| **Section One – Programme Design** |  |
| The advanced career stage preceptorship programme is applicable to pharmacists who have newly started training towards the advanced career stage | ^1-3^ |
| The advanced career stage preceptorship programme is applicable to pharmacists who are returning to advanced career stage after a career break | ^2^ |
| The advanced career stage preceptorship programme is required for pharmacists who are switching from one advanced specialty to another | *RT |
| The start of the preceptorship programme should align with entry into training towards the advanced career stage | ^1^ |
| The advanced career stage preceptorship programme should only be available to pharmacists who are qualified prescribers | *RT ^4-6^ |
| The core learning outcomes for the preceptorship programme should be drawn from the outcomes of the RPS Advanced Pharmacist Curriculum | *RT |
| Any additional agreed learning outcomes should be sourced from alternative specialist frameworks | *RT ^7,8^ |
| An individual learning plan to meet and evidence the learning outcomes should be agreed between the preceptor and the preceptee | ^1^ |
| Completion of a preceptorship programme should take approximately how long?  (participants selected one option that they thought most appropriate) | |
| - 4 months | ^9^ |
| - 6 months | ^10-13^ |
| - 12 months | ^2,3,14-16^ |
| - Flexible depending on the individual | ^1^ |
| The preceptor and preceptee should share a primary workplace (advanced clinical setting) | ^1-3,10-12,14-22^ |
| The preceptor and preceptee should work alongside each other regularly allowing observation of work tasks | ^10,11,17-19,23^ |
| A preceptorship programme should include regular review meetings | ^3,11,13^ |
| A preceptorship programme should include a preceptee training pack | ^2,3,10,15^ |
| A preceptorship programme should include preceptee workshops | ^13^ |
| A preceptorship programme should include preceptee protected learning time | ^13,15^ |
| A preceptorship programme should include preceptor protected learning time | ^22,24^ |
| The employing organisation should ensure preceptor and preceptees protected time. | **NGT |
| A preceptorship programme should include a preceptor policy within your organisation | ^15^ |
| A preceptorship programme should be supported by organisational preceptor support infrastructure | ^11,16,24^ |
| A preceptorship programme should include organisational level preceptor lead | ^14,15^ |
| A preceptorship programme should include a learning contract | ^11,19^ |
| A preceptorship programme should include learning diaries | ^11^ |
| A preceptorship programme should include a requirement for the preceptee to undertake significant event analysis or equivalent | ^11^ |
| A preceptorship programme should include activities designed to induct the preceptee into the advanced practice environment and team | ^1^ |
| A preceptorship programme should include activities designed to integrate pharmacists training towards the advanced career stage into the culture of the advanced practice environment | ^1^ |
| The preceptorship programme must be quality managed by a nominated body | **NGT |
| Preceptors should be able to demonstrate relevant experience at advance practice level | ^2,10,12^ |
| Preceptors should be able to demonstrate competence at the advanced practice level or above in a relevant clinical area | ^3,10,12,25^ |
| Preceptors should be able to demonstrate confidence in the delivery of advanced practice | ^25^ |
| Preceptors should be able to demonstrate relevant experience of teaching clinical practice | ^2^ |
| Preceptors should be able to demonstrate current delivery of clinical practice at the advanced practice level or above | ^18^ |
| The preceptor should be a registered pharmacist | ^2,10,15,17,24^ |
| The preceptor should be any appropriate healthcare colleague | ^14^ |
| In order to undertake the role of preceptor, the individual will need to undertake specific training relating to the programme | ^2,16,26^ |
| Preceptors should not need to undertake specific training other than familiarisation with the advanced pharmacist curriculum and programme materials | ^12,14,15^ |
| Preceptors will need to undertake a regular peer review process of their preceptorship skills | ^18^ |
| Preceptors should be able to demonstrate willingness to undertake the preceptor role | ^2,10,12,15,16,18,23-25^ |
| Preceptors should be able to demonstrate characteristics of a role model | ^2,10,11,15,20,21,23-25^ |
| Preceptors should be able to demonstrate evidence of person-centred practice | ^24^ |
| Preceptors should be able to demonstrate excellent interpersonal skills | ^10-12,15,20-23,25^ |
| Preceptors should be able to demonstrate an understanding of the skill level of pharmacists training towards the advanced career stage | ^1^ |
| Preceptors should be able to demonstrate an understanding of the preceptor/preceptee relationship and the preceptorship programme purpose | ^1,15,22,25^ |
| Preceptees (learners) should be able to demonstrate self-motivation | ^10^ |
| Preceptees should be able to demonstrate active participation | ^10^ |
| Preceptees should be able to demonstrate evidence of person-centred practice | *RT^4^ |
| Preceptees should be able to demonstrate the ability to relate current experience to previous experience | ^10^ |
| Preceptees should be able to demonstrate leadership skills | ^25^ |
| Preceptees should be able to demonstrate initiative | ^25^ |
| Preceptees should be able to demonstrate tolerance of ambiguity | ^25^ |
| Preceptees should be able to demonstrate maturity | ^25^ |
| Preceptees should be able to demonstrate professionalism | **NGT |
| Preceptees should be able to demonstrate good judgement | ^25^ |
| Preceptees should be assessed by review of a portfolio of evidence | ^10,15^ |
| Preceptees should be assessed against competency outcomes | ^2,3,10,15,23^ |
| Preceptees should be assessed for their ability to deliver person centred practice | *RT ^4^ |
| Preceptees should be assessed against completion of supervised learning events | ^2,4,14^ |
| Preceptees should be assessed by submission of reflective account(s) | ^3,4,14^ |
| Preceptees should be assessed by means of a formalised clinical skills assessment appropriate to advanced role | ^18^ |
| Preceptees should be assessed by means of a knowledge test | ^18^ |
| At the end of the Preceptorship programme, support will be continued remotely for example by email or professional conversations. | ^13^ |
| At the end of the Preceptorship programme, support will be continued through standard clinical supervision channels | *RT |

*RT - Added by research team

**NGT – Added by experts during Nominal Group Technique stage

**References**

1. Nursing and Midwifery Council. Principles of Preceptorship. Accessed 10th January 2022, <https://www.nmc.org.uk/globalassets/sitedocuments/nmc-publications/nmc-principles-for-preceptorship-a5.pdf>

2. Black Country Partnership NHS Foundation Trust. Nursing Preceptorship Programme and Portfolio Pack. <https://www.bcpft.nhs.uk/about-us/our-policies-and-procedures/p/1054-preceptorship-nurse-preceptorship-package/file>

3. Lincolnshire Training Hub. Preceptorship Programme for Registered Nurses Working in General Practice. <https://www.lincolnshiretraininghub.nhs.uk/wp-content/uploads/2021/06/LTH-Preceptorship-Programme-2021-a.pdf>

4. Royal Pharmaceutical Society of Great Britain. RPS core advanced curriculum. Accessed September 2022, <https://www.rpharms.com/development/credentialing/core-advanced-pharmacist-curriculum>

5. General Pharmaceutical Council. *Vision 2030*  Accessed 10th January 2022, <https://www.pharmacyregulation.org/sites/default/files/document/gphc-vision-2030.pdf>

6. Royal Pharmaceutical Society Scotland. Pharmacy 2030 a professional vision. <https://www.rpharms.com/Portals/0/RPS%20document%20library/Open%20access/Scotland/Pharmacy%202030%20vision/Pharmacy%202030%20Full%20professional%20vision%20Jan22.pdf?ver=WD2LOOTwG4ejGBfEPC6D0w%3d%3d>

7. NHS Education Scotland. General Practice Clinical Pharmacist Advanced Competency & Capability Framework. <https://learn.nes.nhs.scot/48491>

8. Forsyth P, Warren A, Thomson C, et al. A competency framework for clinical pharmacists and heart failure. *International Journal of Pharmacy Practice*. 2019;27(5):424-435. doi:10.1111/ijpp.12465

9. Forsyth P, Moir L, Speirits I, et al. Improving medication optimisation in left ventricular systolic dysfunction after acute myocardial infarction. *BMJ Open Quality*. 2019;8(3):e000676. doi:10.1136/bmjoq-2019-000676

10. Milwaukee Area Technical College. Paramedic Preceptor Training Program Guidelines. <https://ecampus.matc.edu/Library/SP2018/Paramedic/Paramedic-Preceptor-Guidelines.pdf>

11. Morton-Cooper A, Palmer A. *Mentoring, Preceptorship and Clinical Supervision*. Second Edition ed. Blackwell Science Ltd.; 2000 ISBN 978-0632049677.

12. Yonge O, Billay D, Myrick F, Luhanga F. Preceptorship and Mentorship: Not Merely a Matter of Semantics. *International Journal of Nursing Education Scholarship*. 2007;4(1)doi:doi:10.2202/1548-923X.1384

13. Bungard TJ, Schindel TJ, Garg S, Brocklebank C. Evaluation of a multi staged professional development course for practising pharmacists in anticoagulation management. *The International journal of pharmacy practice*. 2012;20(2):107-17. doi:<https://dx.doi.org/10.1111/j.2042-7174.2011.00171.x>

14. NHS Education for Scotland (NES). Flying Start NHS. <https://learn.nes.nhs.scot/735>

15. North West Region Multi-Professional Preceptorship Community of Practice Group. North West Multi-Professional Preceptorship Framework. <https://www.northerncarealliance.nhs.uk/application/files/7016/4925/2803/Preceptorship_Framework.pdf>

16. Mulherin K, Walter S, Cox CD. National preceptor development program (PDP): Influential evidence and theory. The first of a 3-part series. *Currents in pharmacy teaching & learning*. 2018;10(3):255-266. doi:<https://dx.doi.org/10.1016/j.cptl.2017.12.002>

17. Accreditation Council for Pharmacy Education Chicago I. Accreditation Standards and Key Elements for the Professional Program in Pharmacy Leading to the Doctor of Pharmacy Degree. <https://www.acpe-accredit.org/pdf/Standards2016FINAL.pdf>

18. NES Scotland Deanery. General Practice Trainers. <https://www.scotlanddeanery.nhs.scot/your-development/faculty-development-alliance/resources-for-new-trainers/gptec/>

19. Gurchiek D. The Five Phases of Preceptorship. <https://www.hmpgloballearningnetwork.com/site/emsworld/article/12009234/paramedic-preceptorship>

20. Wolters Kluwer. 9 qualities of effective nursing preceptor programs. <https://www.wolterskluwer.com/en/expert-insights/9-qualities-of-effective-nursing-preceptor-programs>

21. DeAngelis JT, Wolcott MD. A Job Analysis to Define the Role of the Pharmacy Preceptor. *American journal of pharmaceutical education*. 2019;83(7):7196. doi:<https://dx.doi.org/10.5688/ajpe7196>

22. Weitzel KW, Walters EA, Taylor J. Teaching clinical problem solving: a preceptor's guide. *American journal of health-system pharmacy : AJHP : official journal of the American Society of Health-System Pharmacists*. 2012;69(18):1588-99. doi:<https://dx.doi.org/10.2146/ajhp110521>

23. Cuellar LM, Ginsburg DB. *Preceptor's Handbook for Pharmacists*. 4th Edition ed. American Society of Health-System Pharmacists; 2020 ISBN 978-1585286263.

24. The University of Mississipi. Department of Pharmacy Practice - General Information for Preceptor Applicants. <https://pharmacy.olemiss.edu/pharmacypractice/experiential/appe-pharmacy-practice/rxpeppreceptorapplication-pharmacy-practice/#:~:text=In%20the%20context%20of%20experiential,of%20patient%2Dcentered%20pharmacist%20care>.

25. Zahra K, Don H, David H. Desirable Traits of Hospital Pharmacy  Preceptors and Residents. *Canadian  Journal  of Hospital Pharmacy*. 2000;53(No. 5)doi:<https://doi.org/10.4212/cjhp.v53i5.775>

26. NHS Education for Scotland (NES). Educational Supervision in Pharmacy. <https://learn.nes.nhs.scot/56100/pharmacy/programmes/educational-supervision-in-pharmacy>
